# A Toolbox for Optophysiological Experiments in Freely Moving Rats

**DOI:** 10.3389/fnsys.2017.00027

**Published:** 2017-05-11

**Authors:** Stefanie Hardung, Mansour Alyahyay, David Eriksson, Ilka Diester

**Affiliations:** ^1^Ernst Strüngmann Institute gGmbHFrankfurt, Germany; ^2^Faculty of Biology, Albert Ludwig University of FreiburgFreiburg, Germany; ^3^BrainLinks-BrainTools, Albert Ludwig University of FreiburgFreiburg, Germany; ^4^Bernstein Center Freiburg, Albert Ludwig University of FreiburgFreiburg, Germany

**Keywords:** optogenetics, extracellular recordings, rat model system, electrode/wire arrays, microdrives

## Abstract

Simultaneous recordings and manipulations of neural circuits that control the behavior of animals is one of the key techniques in modern neuroscience. Rapid advances in optogenetics have led to a variety of probes combining multichannel readout and optogenetic write in. Given the complexity of the brain, it comes as no surprise that the choice of the device is constrained by several factors such as the animal model, the structure and location of the brain area of interest, as well as the behavioral read out. Here we provide an overview of available devices for chronic simultaneous neural recordings and optogenetic manipulation in awake behaving rats. We focus on two fixed arrays and two moveable drives. For both options, we present data from one custom-made (in house) and one commercially available device. Here we provide evidence that simultaneous neural recordings and optogenetic manipulations are feasible with all four tested devices. Further we give detailed information about the recording quality, and also contrast the different features of the probes. As we provide detailed information about equipment and building procedures for combined chronic multichannel readout and optogenetic control with maximum performance at minimized costs, this overview might help especially researchers who want to enter the field of *in vivo* optophysiology.

## Introduction

A detailed understanding of neural circuits that control the behavior of animals requires the simultaneous recording of multiple neurons and manipulations of those circuits in freely moving animals. Electrophysiological recordings have long been used to gather information about neural activity, often in combination with electrical stimulation or pharmacological manipulations (Miller and Wilson, 2008). Optogenetics, a more recent technique, allows the rapid, reversible within-trial manipulation of distinct brain areas without damaging connections to other areas. Thus, the combination of both techniques, electrophysiology and optogenetics, allows for simultaneous manipulation of interwoven network hubs while monitoring the activity of neuronal populations (Buzsáki et al., 2015; Dufour and De Koninck, 2015). Here, we systematically analyzed two general types of neural probes combining electrophysiological recordings with optical hardware in awake behaving rats: (1) chronically fixed arrays [custom-made optrode-MWA and Microprobes-MEA (Microprobes, Gaithersburg, MD, USA)] and (2) screw-based microdrives [Optetrode (Anikeeva et al., 2012) and VersaDrive-8 Optical (Neuralynx©, Bozeman, MT, USA)]. While fixed devices can place many electrodes into different brain areas, common microdrives can only target one brain area since they are too large to be implanted at multiple locations on a rat’s skull (Haiss et al., 2010). The major advantage of microdrives is that they allow advancing electrodes down into the animal’s brain on a micrometer scale, even after implantation (Haiss et al., 2010). We contrast all devices in terms of recording quality, longevity, convenience in handling, and interference with animal behavior. Lastly, we offer detailed protocols for the fabrication and implantation of each device. With this, we provide a toolbox for a chronic multichannel readout combined with optogenetic control with maximum performance at minimized costs, tailored to the neuroscientific question at hand and ideally suited for rats.

## Materials and Methods

### Animals

Male adult CD^®^ IGS rats (Sprague–Dawley, 6–8 weeks of age, 350–500 *g*, in-house bred) were employed in this study. They were pair-housed (2 per group) in large individually ventilated cages under a 12 h light dark cycle (light off from 8 a.m. to 8 p.m., time span of training and experiments). This study was carried out in accordance with the recommendations of the guidelines RL 2010 63 EU, Regierungspräsidium Darmstadt and Freiburg. The protocol was approved by the Regierungspräsidium Darmstadt and Freiburg.

### Plasmid DNA Amplification and Virus Production

The plasmid (rAAV5-hSyn-eNpHR3.0-eYFP) was provided by courtesy of Karl Deisseroth’s Lab (Stanford University). We directly transfected plasmid-DNA into chemically competent Stbl3 *E. coli* cells (Invitrogen, Darmstadt, Germany) in order to amplify the plasmid containing the eNpHR3.0 construct. The vector possessed an ampicillin resistance. Therefore, transfected Stbl3 cells were transferred on Lysogeny broth (LB)-Agar with ampicillin (100 mg/ml) (Carl Roth GmbH, Karlsruhe, Germany) and incubated over night at 37°C. On the next day, we picked one colony and transferred it into 250 ml liquid LB-medium with 100 mg/ml ampicillin. After overnight incubation, we harvested bacterial cells by centrifugation at 4000 rpm for 15 min at 4°C and purified the plasmids with the Qiagen EndoFree Plasmid Maxi Kit (Qiagen, Hilden, Germany) according to the manufacturers’ protocol. We sent 300 μg of amplified plasmid for AAV vector production (serotype 2 pseudotyped with serotype 5) to the viral vector core at the University of North Carolina (UNC Vector Core, Chapel Hill, NC, USA) and received viral vectors with a titer of 4.0 × 10^12^ genome copies (g.c.) ml^-1^.

### Fabrication of Fixed Optrode-Multi-Wire Arrays (Optrode-MWAs)

**FIGURE 1:**
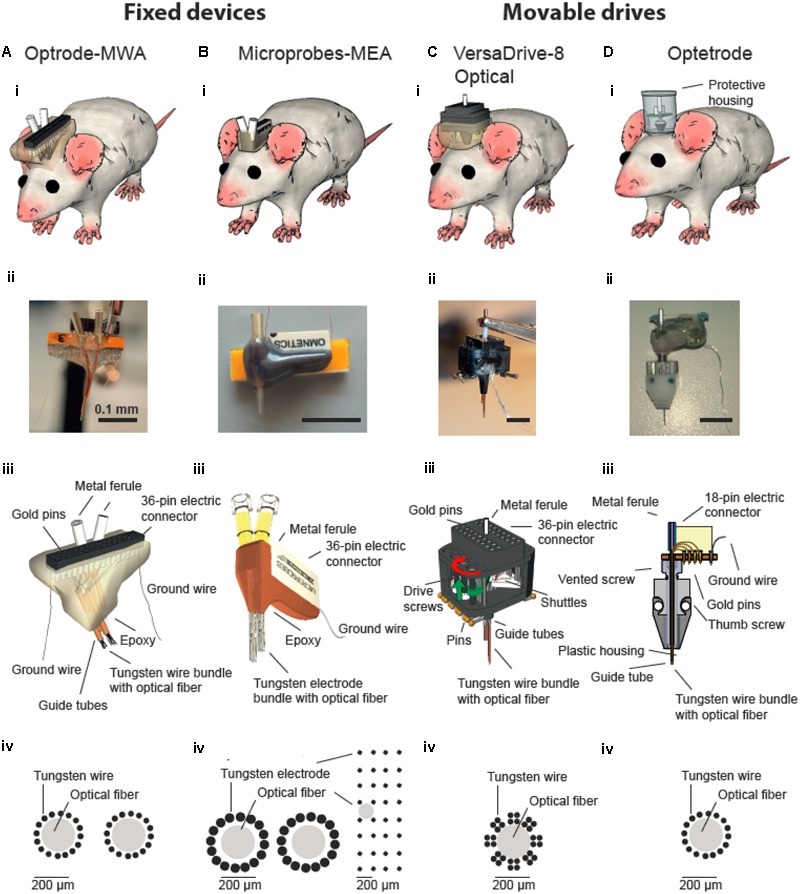
**Multichannel devices for simultaneous *in vivo* neural recordings and optical manipulations.** Optical fibers are incorporated into either fixed **(A,B)** or movable multichannel devices **(C,D)**. **(i)** Illustration of chronically implanted devices in rats. Photographs **(ii)** and schematic drawings **(iii)** of assembled multichannel arrays. **(iv)** Schematic drawing of wires/electrodes and optical fibers arrangements. **(Biv)** Two configurations of Microprobe-MEA were used targeting either two structures in the medial prefrontal cortex (left) or a larger superficial area in motor cortex (right). **(Biii–Diii)** were modified with permission from (Anikeeva et al., 2012) and (Microprobes for Life Sciences, Gaithersburg, MD, USA; Neuralynx©, Bozeman, MT, USA).

**FIGURE 2:**
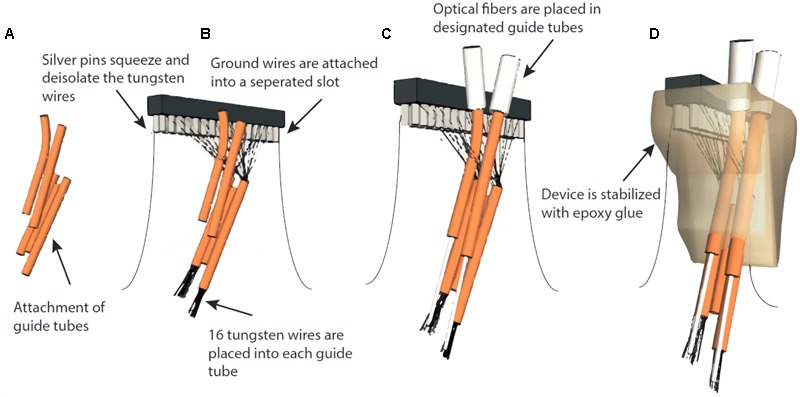
**Assembly of fixed optrode-MWAs. (A)** AWG guide tubes were combined with superglue. **(B)** Tungsten wires were placed into the guide tubes and placed into separate slots of a Conn Strip Socket, followed by the insertion of a metal pin that squeezed and de-insulated the tungsten wire, thus, connecting pins and wires. Ground wires were de-insulated and placed into designated slots and fixed with silver pins. **(C)** The optical fibers were placed into the respective guiding tubes. **(D)** To make the entire array stable, we fixed the wires to a Conn Strip Socket and guide tubes with epoxy glue.

For simultaneous optical manipulation and electrophysiological recordings, we designed probes for targeting either motor cortex (M1, *n* = 4) or two PFC subareas (*n* = 4). Arrays for M1 stimulation and recordings contained one 200 μm fiber (0.37 NA, Doric lenses, Quebec, QC, Canada) and 32 tungsten wires (outer diameter 35 μm, 200 to 600 kOhm impedance, polyimide insulation, WHS Sondermetalle, Grünsfeld, Germany). To enable stimulation and simultaneous recordings in two PFC subareas, we further fabricated optrode-MWAs with two separated bundles each containing one 200 μm fibers and 16 tungsten wires (**Figures 1Ai–iv** and **Tables 1, 2**, 1st column). We cut 5 segments of 30-AWG guide tubes (Small Parts, Logansport, IN, USA) and combined them with superglue (UHU GmbH & Co KG, Bühl/Baden, Germany) (**Figure 2A**). We placed 16 single tungsten wires (outer diameter 35 μm) into each of the 30-AWG guide tubes for one PFC subarea and led each wire into one separate slot of a Conn Strip Socket (0.0″, Digi-Key, Thief River Falls, MN, USA), followed by the insertion of a metal pin that squeezed and de-insulated the tungsten wire (0.05″, Digi-Key, Thief River Falls, MN, USA), thus, connecting pins and wires. For each cortical area, an additional wire was de-insulated at the tip by application of heat and placed into one guide tube. The de-insulated wires represented the reference wires. Furthermore, two additional silver wires [de-insulated diameter 125 μm, Science Products (Hofheim, Germany)], which functioned as electrical grounds, were also de-insulated at their tips by application of heat. The grounds were not placed into the AWG guide tubes, but directly into designated slots and were afterward fixed by pushing a silver pin into the same slot (**Figure 2B**). The optical fibers for optogenetic manipulations were positioned into the respective guiding tube (**Figure 2C**). To stabilize the entire array, we fixed the wires to the combination of Conn Strip Socket and guide tubes with epoxy glue (Messinger Schrauben GmbH, Frankfurt, Germany, **Figure 2D**). After the epoxy had cured, we cut the wires to the required lengths. The grounds were cut to a length of 7 cm. We measured the impedance of each electrode wire in saline solution (B. Braun Melsungen AG, Melsungen, Germany) with an impedance meter (Model IMP-2AMC 18 Channel Impedance Tester, Microprobes, Gaithersburg, MD, USA) and marked broken channels.

**Table 1:** Materials and costs.

	CUSTOM-MADE OPTRODE MWA	MICROPROBES OPTO-MEA	VERSADRIVE-8 OPTICAL	OPTETRODE
*Price per array or drive* (*total*) [€]	~50.00	1292.00	258.04	~180.00
*References*	https://www.optophysiology.uni-freiburg.de/	https://microprobes.com/	http://neuralynx.com/	Anikeeva et al., 2012
**REQUIRED MATERIAL**
*VersaDrive 8 Optical*			1022.83/5pcs	
*Optical fiber* (*Doric lenses*)	23.40/pc	–	37.00/pc	37.00/pc
*Guide tubes* (*Small Parts*)	AWG 30 (1.90/in)	–	AWG 28 (~0.73/in)	AWG 24 (0.32/in)
*EIB-18* (*Neuralynx*)	–	–	–	81.25/pc
*EIB Gold Pins* (*Neuralynx*)	–	–	–	85.24/500pcs
*Tungsten wire* (*WHS Sondermetalle*)	10.60/meter	–	10.60/meter	10.60/meter
*Conn Strip Socket* (*0.05″, Digi-Key*)	8.95/pc	–	–	–
*Epoxy glue* (*Messinger Schrauben*)	5.14/pc	–	5.14/pc	5.14/pc
*Vented screw* (*RC-Schrauben*)				0.73/pc
*Modification of vented screw* (*ESM Erodier Service Mueller GmbH*)				0.50/pc
*Protecting housing* (*Metallverkaufsgesell-schaft mbH*)				~30.00/pc
*Thumbscrew* (*Metallverkaufsgesell-schaft mbH*)				~2.00/pc
*Plastic housing*				~5.00/pc
*Interference pins*				~1.00/pc

**Table 2:** Features of the tested MWA/MEA devices.

	CUSTOM-MADE OPTRODE MWA	MICROPROBES OPTO-MEA	VERSADRIVE-8 OPTICAL	OPTETRODE
*# Electrodes*	32	32	32	16
*# Fibers*	2	2	1	1
*# Simultaneously targetable Areas*	2	2	1	1
*Spatial Precision*	Limited spacing precision	Custom spacing between electrodes is in the range of 0.1–1 mm	0.51 mm between each electrode bundle (or tetrode)	Limited spacing precision
*Optical fibers*	MF2.5	MF2.5	MF1.25	MF1.25
*Electrode or wire material (other materials are possible)*	Tungsten	Tungsten	Tungsten	Tungsten
*Electrode diameter*	35 μm coated diameter	75 μm coated diameter	35 μm coated diameter	35 μm coated diameter
*Connector*	MILL-MAX	Omnetics	MILL-MAX	Omnetics

### Fabrication of Fixed Optrode-Multi-Electrode Arrays (Optrode-MEAs) and Movable Drives (VersaDrive-8 Optical and Optetrode)

Optrode-MEAs were individually designed and ordered from Microprobes, Gaithersburg, MD, USA (**Figures 1B–D** and **Tables 1, 2**, 2nd column**)**. The Optetrode (Anikeeva et al., 2012) and VersaDrive-8 Optical (Neuralynx©, Bozeman, MT, USA) were assembled according to the published protocols [see Anikeeva et al., 2012 and Neuralynx^1^ (accessed December 13, 2016), respectively]. However, please note that we used single electrode wires instead of tetrodes (**Figures 1C,D** and **Tables 1, 2**, 3rd and 4th column).

### Stereotactic Injection and Implantation

For electrophysiological recordings and optogenetic manipulations, we implanted multi wire arrays (MWA) and multi electrode arrays (MEA) in cortical areas of 18 rats (custom-made optrode MWA: 7, Microprobes opto-MEA: 3, VersaDrive-8 Optical: 3, Optetrode: 5, see **Table 3** for data of individual rats). The animals received inhalative gas anesthesia with isoflurane (CP-Pharma, Burgdorf, Germany) with O_2_ as carrier gas. We induced anesthesia with 75 mg/kg Ketamine (Medistar, Holzwickede, Germany) + 50 μg/kg Medetomidine (Orion Pharma, Espoo, Finland) in isotonic saline injected intraperitoneal (i.p.) and maintained animals anesthetized at 2 % isoflurane and 0.5 l/min O_2_. Additionally, we subcutaneously (s.c.) administered pre-surgery 0.05 mg/kg Buprenorphine as analgesic (Selectavet Dr. Otto Fischer GmbH, Weyarn/Holzolling, Germany) and 0.4 ml/kg Baytril as antibiotic (Bayer Health Care, Leverkusen, Germany). During the whole course of the surgery, we placed the rats on a warming pad and continuously monitored their temperature by using a rectal sensor. The eyes were covered with ophthalmic ointment (Bepanthen, Bayer Health Care, Leverkusen, Germany). To ensure the rats’ fluid balance, we injected 5 ml saline solution (s.c., 0.9 %, B. Braun Melsungen AG, Melsungen, Germany). Using a sterile cotton tip, we cleaned the surgical site of the head in 3 repetitions with Braunol (B. Braun Melsungen AG, Melsungen, Germany) followed by Kodan (Schülke, Norderstedt, Germany). After opening the skin and removing the tissue with a bone scraper until the stereotactic landmarks lambda and bregma were clearly visible, we leveled the head; i.e., the head was placed in a stereotaxic frame (World Precision Instruments, Sarasota, FL, USA) in a way that the difference in height between bregma and lambda was <0.05 mm. Craniotomies of ~1.5 mm in diameter were made bilaterally above the selected brain areas. We injected 1 μl of the viral vector rAAV5-hSyn-eNpHR3.0-eYFP with a rate of 100 nl/min into the respective subareas with a 10 μl gas-tight Hamilton syringe (World Precision Instruments, Sarasota, FL, USA). To minimize the reflux of the injected volume, we left the injection needle in the tissue for additional 10 min before slowly retracting it from the brain. For stabilization of the implants, we added miniature self-tapping screws (J. I. Morris Company, Southbridge, MA, USA) to the skull. MWA were placed into a holder and slowly lowered to the implantation site. We wrapped the de-insulated ground wires around a separate skull screw and carefully lowered electrodes into the brain to the designated locations. Finally, the implants were fixed to the animal’s head by applying several layers of dental cement. For this purpose, we protected the brain surface from dental cement by applying a thin layer of Vaseline (Bombastus-Werke AG, Freital, Germany) onto the brain around the implant. Afterward, we applied a thin layer of super bond C&B cement (Sun Medical Co., LTD, Moriyama City, Shiga, Japan) onto the skull and implant, and added several layers of Paladur (Heraeus, Hanau, Germany) to stabilize everything. The surgical site was closed around the implant by interrupted sutures with 5-0 silk (SMI AG, St. Vith, Belgium) and the rats were treated for 5 days post-surgery with 0.4 ml/kg Baytril s.c.

**Table 3:** Summary of neural recordings from all implanted animals.

ANIMALS	RECORDING DURATION IN DAYS	TOTAL NUMBER OF RECORDED SINGLE UNITS	TOTRAL NUMBER OF RECORDED MULTI UNITS	ACTIVE ELECTRODES [%]^∗^	ELECTRODES WITH MORE THAN ONE SINGLE UNIT [%]^∗∗^
**OPTRODE-MWA**
*O- MWA rat 1*	7	15	47	35.14 (52/148)	3.8 (2/52)
*O- MWA rat 2*	7	27	45	31.52 (58/154)	6.9 (4/58)
*O- MWA rat 3*	6	43	46	45.83 (66/144)	10.6 (7/66)
*O- MWA rat 4*	11	41	70	53.59 (97/181)	3 (3/97)
*O- MWA rat 5*	16	109	66	58.68 (142/242)	9.8 (14/142)
*O- MWA rat 6*	12	31	57	55.59 (85/152)	1.1 (1/85)
*O- MWA rat 7*	7	67	24	60.05 (72/119)	20.8 (15/72)
**Microprobes**
*M- MEA rat 1*	10	23	19	29.91 (35/117)	8.5 (3/35)
*M- MEA rat 2*	10	46	3	47.14 (33/70)	33.3 (11/33)
*M- MEA rat 3*	9	4	6	24.32 (9/37)	0 (0/9)
**Optetrode (rats)**
*Optetrode rat 1*	42	46	28	22.04 (67/304)	5.9 (4/67)
*Optetrode rat 2*	19	13	19	22.22 (32/144)	0 (0/32)
*Optetrode rat 3*	15	16	10	17.48 (25/143)	4 (1/25)
*Optetrode rat 4*	15	5	19	19.82 (22/111)	0 (0/22)
*Optetrode rat 5*	15	2	12	16.25 (13/80)	0 (0/13)
**Optetrode (mouse)**
*Optetrode mouse 1*	7	18	2	35.42 (17/48)	17.6 (3/17)
**Versadrive-8 optical**
*VD-8 opt rat 1*	24	219	228	86.05 (296/344)	19.9 (59/296)
*VD-8 opt rat 2*	23	82	169	50.46 (165/327)	15.6 (33/165)
*VD-8 opt rat 3*	24	152	179	67.63 (211/312)	9.7 (16/211)

*^∗^Calculated by dividing the number of channels with neural activity by the total number of all electrodes (excluding initially broken electrodes). ^∗∗^Calculated by dividing the number of channels with more than one single unit by the number of electrodes with neural activity.*

### Optogenetic Manipulation and Electrophysiological Recordings

We started optogenetic experiments 6 weeks after opsin injection and MWA implantation. All implanted devices were equipped with either one (VersaDrive-8 Optical and Optetrode) or two (custom-made optrode-MWA and Microprobes opto-MEA) 200 μm fibers (0.37 NA, Doric lenses, Quebec, QC, Canada). Optical fibers were coupled to the LightHUB compact laser combiner (OMICRON Laserage, Rodgau-Dudenhofen, Germany) via 2 mono fiberoptic patchcords (Doric lenses, Quebec, QC, Canada) and a fiber-optic rotary joint (FRJ_1x2i_FC-2FC, Doric lenses, Quebec, QC, Canada). Optogenetic inhibition was achieved with yellow laser light (λ = 594 nm, 254 mW/mm^2^ to 318 mW/mm^2^ light power density, measured at the tip of a 200 μm diameter fiber). We recorded neural activity from each implanted animal with a 32-channel data acquisition system [Tucker–Davis Technologies (TDT), Alachua, FL, USA] and filter settings of 300 Hz for high-pass and 5,000 Hz for low-pass filtering. Neural activity following optical inhibition was compared to the baseline activity by sliding-window paired-sample *t*-tests (bin size 100 ms, *p* < 0.05).

### Spike Sorting

**FIGURE 3:**
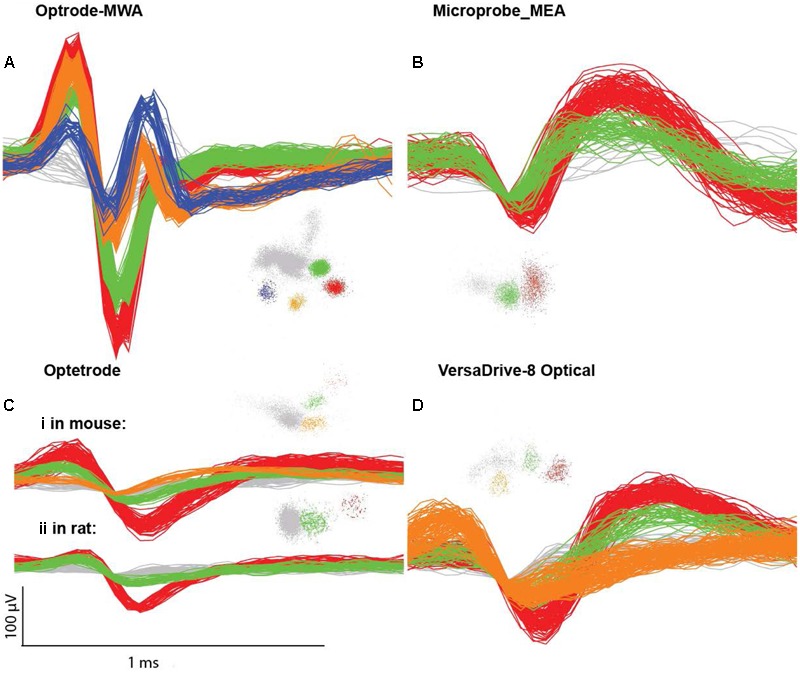
**Single unit examples. (A)** Waveforms and clusters from an optrode MWA, **(B)** Microprobes MEA, **(C)** Optetrode **(i)** from a mouse and **(ii)** from a rat, **(D)** VersaDrive 8 Optical. Colored clusters represent hand sorted units visualized in PC1 versus PC2 feature space (Plexon OFS). The noise waveforms and clusters are marked in gray.

We acquired neural data with a sampling frequency of *F*s = 24,414.0625 Hz and sorted neural units offline using the Plexon Offline Sorter (OFS) (Plexon Inc., Dallas, TX, USA) and Wave_clus (Quiroga et al., 2004). A Matlab algorithm was used for artifact removal, thereby processing data for automatic waveform clustering (Quiroga et al., 2004). The entire algorithm detected and classified neural signals in three steps: (1) artifact removal and spike detection, (2) extraction of distinctive features from the spike shapes (waveform and corresponding time stamps), and (3) clustering of the selected spike features. Detected waveforms and corresponding time stamps were clustered by using the Wave_clus algorithm. We verified the reliability of the automatic spike sorting by manually sorting individual channels with the OFS (Plexon Inc., Dallas, TX, USA). We sorted the raw data by using the semi-automatic Valley-Seeking method and manually refined the clusters (Zhang et al., 2007). To evaluate the quality of given clusters, we ensured good separation from the noise level as well as additionally recorded neural activity and calculated the interspike interval (ISI) for each modulated cluster (see **Figure 3** for sorting examples). The ISIs were used to define single unit clusters with ISIs smaller than 1.0 ms occurring at a frequency of less than 1 %. Furthermore, the waveform of single units had to be constant for the entire recording time. Both, single unit activities as well as multi-unit activities were used for the purpose of this study.

### Assessing the Quality of Electrophysiological Recordings

To analyze the quality of the electrophysiological recordings, we applied three independent measures: For each recording day, we computed and statistically compared the following measures with a n-way analysis of variance (ANOVA) followed by multiple comparison tests (with Bonferroni correction): (1) the percentage of active electrodes. An electrode was defined as active if we detected neural activity during the recording session. If an electrode never showed neural activity throughout all the recording sessions, the electrode was removed from this analysis as well as all further analyses. (2) The ratio of number of units to the number of active electrodes. (3) The signal to noise ratio (SNR) for each active channel. We defined the signal as the peak amplitude of the unit and the noise as the mean noise level. Additionally, to compare the data quality before and after the wires were lowered in the brain, we computed SNR move triggered averages for movable drives (VersaDrive-8 Optical). The move triggered average was calculated as the average of the SNR for all electrodes of the respective probe on the day of the move compared to the average of the day before and the averages of five days after the drive move.

## Results

We recorded extracellularly from 18 rats with four different probes during freely moving behavior (Hardung et al., 2017). We implanted 10 rats with fixed arrays (seven rats with optrode MWA, three rats with Microprobes opto-MEA) and eight rats with movable drives (five rats with optetrode, and three rats with VersaDrive-8 Optical). Recordings started after a week of recovery post surgery (**Table 3** and **Figure 3**).

### Neuronal Recordings with Fixed Arrays

Optrode-MWA were assembled in house (**Figures 1Ai–iv**). We implanted the optrode-MWA either in motor cortex (**Table 3** O-MWA rat 1–5) or in prelimbic and infralimbic prefrontal cortex (**Table 3** O-MWA rat 6–7). We recorded an average of 8.4 sessions (±1.26 SEM) and a total of 333 single units and 355 multi units. In all rats with an optrode-MWA, we were able to isolate more than one single unit per electrode in 46 cases over a course of 38 recording sessions. These cases were focused on a small subset (8 %) of active electrodes (46 electrodes out of 572 active electrodes where each electrode was counted as unique data point for each recording session, see **Table 3** for individual data of each rat). Further, we implanted 3 rats with Microprobes opto-MEAs (2 in motor cortex and 1 in prelimbic cortex). We recorded from these three rats for 7.3 sessions (±0.3 SEM) with a total of 73 single units and 28 multi units. In 2 of the 3 rats, we were able to isolated more than one single unit per electrode in 14 cases over the course of 13 recording sessions, resulting in 18.1 % (14/77) of the active electrodes. In 5 of 10 rats which were implanted with fixed arrays, we recorded for ≥10 days (16 days for O-MWA rat 5). The high longevity of the probes in these five rats was most likely due to a lower spatial density of electrodes, which minimized the tissue damage around the electrode tips.

### Neuronal Recordings with Movable Drives

**FIGURE 4:**
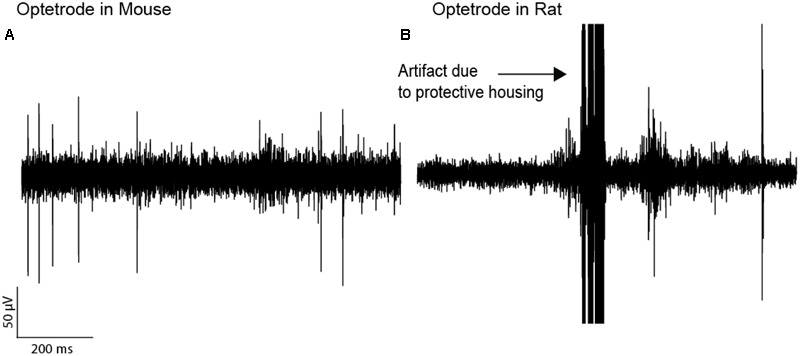
**Optetrode recordings are more stable in mice than in rats. (A)** A trace of electrophysiological recordings in a mouse implanted with an Optetrode drive. **(B)** Recordings in a rat with the Optetrode drive surrounded by the protective housing. The arrow indicates the artifact which were caused by the rat hitting the metal wall with the protective housing when entering the reward port.

We further tested whether optetrodes are suitable for rats (Anikeeva et al., 2012). When implanting optetrode in rats, we faced two issues: (1) given that rats are 20 times as big as mice and thus much stronger, they were able to rip off the probes within seconds. To overcome this problem, we built a protective aluminum housing around the probe (**Figure 1Di**). With this protective housing, we were able to prevent mechanical destruction for more than a month. However, the housing caused a second problem: (2) noise artifacts occurred whenever the housing was in contact with any metallic part of the behavioral chamber. Hence most of the neural activity in the recordings were masked by the artifacts (**Figure 4B**) and because of this, we observed a lower number of single units (82) and multi units (88) with the optetrode in rats even when we recorded for as long as 46 days. In line with this, we were only occasionally able to isolate more than one single unit per electrode, i.e., five times over 55 recording sessions (3.1 %, 5 electrodes out of 159 active electrodes, again each electrode was counted as unique data point for each recording session, **Table 3**, Optetrode rat 1). To contrast our findings in rats, we implanted an optetrode in a mouse (**Figure 4A**). Here, we were able to isolate 18 single and two multi units in three recording sessions over 7 days. We further isolated more than one single unit per electrode three times over the course of 3 recording session. Additionally, we implanted 3 rats with the commercially available VersaDrive-8 Optical probes. Over an average of 22.6 sessions (±0.3 SEM), we isolated 453 single units and 576 multi units in all three rats. Further, we successfully isolated more than one single unit per electrode over a course of 37 recording sessions in 16.07 % of the electrodes (108 electrodes out of 672 active electrodes, **Table 3**).

### Quantifying the Quality of the Recordings

**FIGURE 5:**
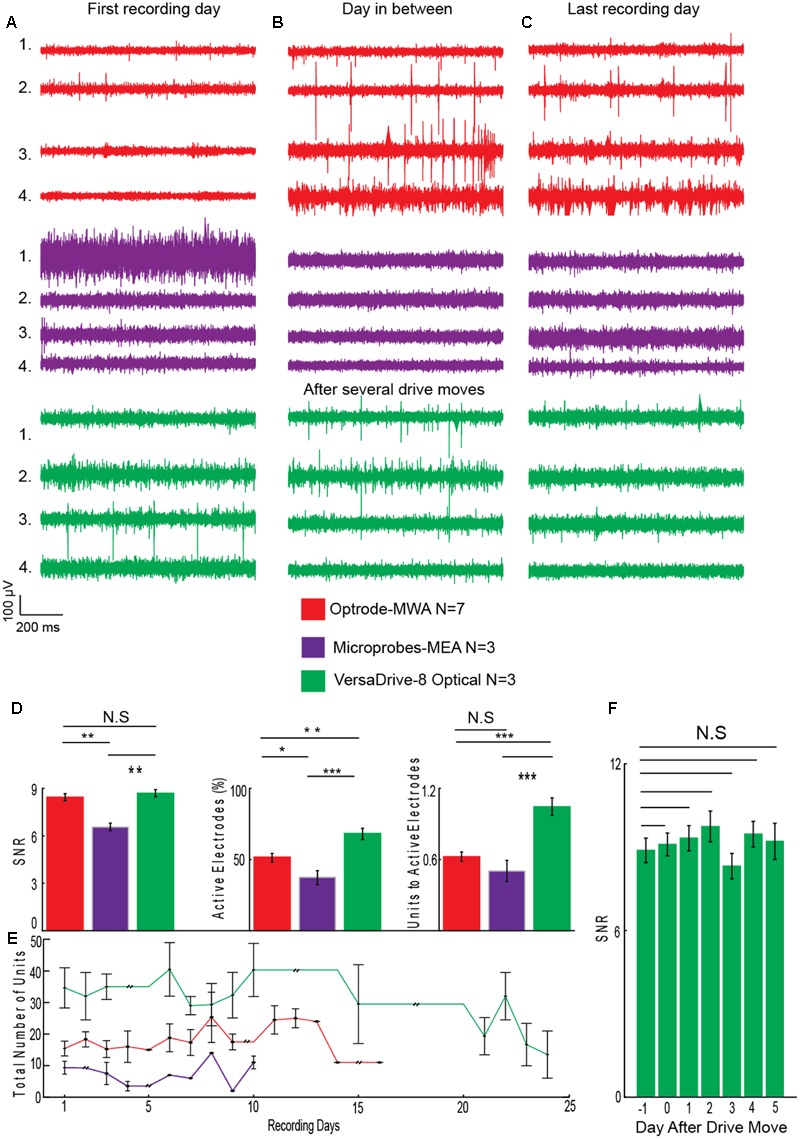
**Assessment of recording quality. (A)** Single channel examples of extracellular recordings from the first recording day, **(B)** from a day several days after implantation, and **(C)** from the last day of recordings before experiments were finished. **(D)** Quality analyses for optrode-MWA (16 recording days), Microprobes MEA-optical (10 recording days), VersaDrive-8 optical (24 recording days): SNR (left panel), percentage of active electrodes (middle panel), and ratio of units to active electrodes (right panel). **(E)** Sum of all units recorded with each probe during each recording day (aligned to the first recording day) averaged across animals. Double black lines represent periods without neural recordings. **(F)** SNR of VersaDrive-8 Optical on days before and after lowering the drive in the brain. Red: optrode-MWA, purple: Microprobes-MEA, and green: VersaDrive-8 Optical. Detailed information about the number or recorded units and active channels for each probe is listed in **Table 3**. N.S: *p* > 0.05, ^∗^*p* < 0.05, ^∗∗^*p* < 0.01, ^∗∗∗^*p* < 0.001, *n*-way ANOVA; error bars, SEM.

In total we recorded the following numbers of sessions and units per probe type: Optrode-MWA: 688 units, 572 active electrodes (7 rats and 66 sessions), Microprobes: 101 units, 77 active electrodes (3 rats and 29 sessions), VersaDrive: 1029 units, 672 active electrodes (3 rats and 71 sessions). To assess the quality of the recordings, we averaged the number of recording sessions per probe (8.4 sessions ±1.26 SEM for the optrode-MWA, 7.3 sessions ±0.3 SEM for Microprobes-MEA, and 22.6 sessions ±0.3 SEM for VersaDrive-8 optical) and applied the following three measures. (1) SNR (signal and noise were defined as the mean of the amplitude of the largest unit on the electrode and the mean noise level, respectively). (2) Percentage of active electrodes (excluding broken wires). (3) Ratio of total units to the number of active electrodes. Given the issues with Optetrode in rats, we opted to exclude the Optetrode implants from further analyses. For future applications, we suggest a different material for the protective housing (e.g., rat teeth proof hard plastic). Prior to quantifying the quality of the data, we observed that in some cases it took several days before isolation of single units became feasible (**Figures 5A–C** electrodes 2 and 3 for the optrode-MWA and electrodes 1 and 2 for the VersaDrive-8 Optical, red and green traces, respectively). However, there was no significant difference between the SNR on the first recording day compared to later recordings sessions (*p* > 0.05, *n*-way ANOVA). To compare the SNR across probes, we averaged the SNR for all recording days across all implanted rats for each probe. With this approach, we observed no significant difference between optrode-MWA (mean: 8.34 ± 0.224 SEM) and VersaDrive-8 Optical (mean: 8.69 ± 0.219 SEM, *p* > 0.05), while the SNR was significantly lower for the Microprobes optical-MEA (mean: 6.55 ± 0.239 SEM) compared to optrode-MWA and VersaDrive-8 Optical (*p* < 0.01, **Figure 5D** left subpanel). Moreover, the percentage of active channels was significantly lower in the Microprobes Optical-MEA (77 active electrodes out of 224 electrodes recorded from three rats, mean: 37.60 ± 4.947 SEM) compared to optrode-MWA (572 active electrodes out of 1108 electrodes recorded from seven rats, mean: 51.64 ± 3.090 SEM, *p* < 0.05) and VersaDrive-8 optical (672 active electrodes out of 983 electrodes recorded from three rats, mean: 68.37 ± 3.906 SEM, *p* < 0.001, **Figure 5D** middle subpanel). Further, the percentage of active channels in VersaDrive-8 Optical was significantly higher than in the optrode-MWAs (*p* < 0.01). Finally, the ratio of units to active channels was significantly higher in the VersaDrive-8 Optical (mean: 1.045 ± 0.073 SEM) compared to optrode-MWA (mean: 0.624 ± 0.088 SEM, *p* < 0.001) and Microprobes optical-MEA (mean: 0.504 ± 0.088 SEM, *p* < 0.001, **Figure 5D** right subpanel). To further assess the quality of the recordings, we calculated the total number of units for each probe and each recording session averaged across animals (**Figure 5E**). Interestingly, the total number of units did not differ significantly within each array type. Instead, the number remained relatively constant over time (*p* > 0.05, paired-sample *t*-test). The lowest number of sortable units was received with the Microprobe-MEA. Since the overall yield was rather low with this device, we stopped recordings after maximally 10 days during which we recorded on average for 7.3 sessions ±0.3 SEM. The Optrode-MWA reached a low plateau around day 16 and 8.4 recording sessions ±1.26 SEM while the yield of the VersaDrive-8 Optical dropped to a comparable level after 24 days and on average 22.6 recording sessions ±0.3 SEM (**Figure 5E**). Furthermore, we assumed that the recording quality improves after electrodes were advanced via the moveable drives. Surprisingly, we did not observe any significant change after lowering the drive in the brain (**Figure 5F**, *p* > 0.05, *n*-way ANOVA). This might have been due to the pressure on the brain tissue which was evoked when the electrodes as well as the large fiber were moved down collectively.

### Simultaneous Optogenetic Manipulation and Electrophysiological Recordings

**FIGURE 6:**
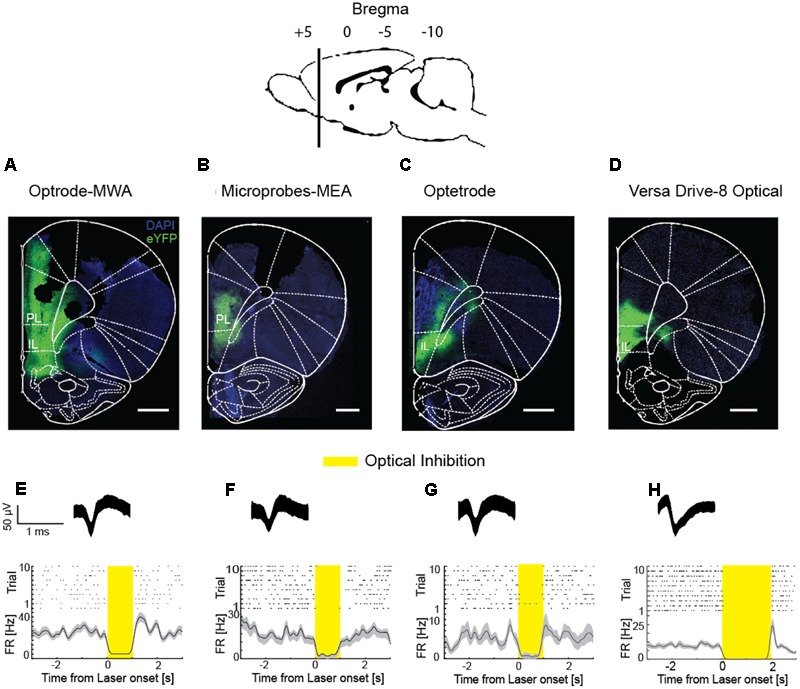
**Simultaneous extracellular recordings and optical inhibition. (A–D)** Coronal slices through rat prefrontal cortices including sites expressing the fusion protein halorhodopsine-eYFP. Scale bar = 1 mm. **(E–H)** Raster plots and PSTHs of single units which were optically suppressed. Insets on top represent waveforms of the respective units. First column: Optrode-MWA, second column: Microprobes-MEA, third column: Optetrode, fourth column: VersaDrive-8 Optical. Shaded areas – SEM. Bin size: 10 ms.

The distance between the fiber and the electrodes plays a crucial role in simultaneous recordings and stimulation. On the one hand, the electrode-fiber distance should be minimal to maximize the number of neurons which get stimulated. On the other hand, neurons close to the fiber site are likely to be damaged due to the lesion caused by the fiber (Cardin et al., 2010). Thus, a few hundred micrometers distance between the fiber and the nearest electrode are ideal. In all tested probes, we recorded and stimulated in both, deep structures (i.e., 5.6 mm from the brain’s surface **Figures 6A–D**) as well as superficial structures (i.e., 1.5 mm from the brain’s surface). Moreover, we were able to record single and multi-unit activity and simultaneously stimulate optically (**Figures 6E–H**) in freely moving rats. Each rat received 1 μl of the viral vector rAAV5-hSyn-eNpHR3.0-eYFP into the respective cortical subarea (PL, IL, or motor cortex). After 6 weeks of viral expression, we stimulated the previously injected area with yellow laser light (pulse duration: 1–2 s, λ = 594 nm, 254 mW/mm^2^ light power density, measured at the tip of a 200 μm diameter fiber) while simultaneously performing neural recordings in the same area. In all implantable probes, optical inhibition resulted in a significantly decreased spiking activity compared to the baseline activity (**Figures 6E–H**, *p* < 0.05, sliding-window paired-sample *t*-test), thus providing evidence that all four devices are suitable for stable light delivery. In detail, we measured significantly reduced activity in reaction to the light with the optrode-MWA in 22 out of 35 units (62.86%). Further, we successfully inhibited 5 out of 17 units (29.41%) with the Microprobes-MEA. With the Optetrode, we successfully inhibited 5 out of 15 units (33.33%). Finally, we successfully inhibited 8 out of 16 units (50%) with the VersaDrive-8 Optical.

## Discussion

Here we give an overview of available devices for chronic simultaneous neural recordings and optogenetic manipulation in awake behaving rats. We focused on two available types of array: (1) fixed (*n* = 2) and (2) movable devices (*n* = 2). We provided evidence that all four tested neuroprobes are suitable for simultaneous neural recordings and optical manipulations. However, depending on the research question and animal model, a specific probe might be favorable over the others. From an economic perspective, the custom-made optrode-MWA are the most affordable solution and thus might be a good starting point for pilot experiments. Further, they are relatively easy to build with standard lab equipment, again making them ideally suitable for first tests. Additionally, a variety of configurations can be created, i.e., even simultaneous targeting of multiple brain areas is feasible (we tested up to two brain areas at a time with two fibers and 32 electrodes but more fibers and electrodes are feasible as well), thus making these arrays also attractive for more than pilot experiments. One obvious drawback is the fixed nature of the arrays: if the targeted depth turns out to be not ideal, the recording wires cannot be moved to a different depths afterward. The Microprobes-MEA are fixed as well, and thus cannot be moved after the implantation either. However, they offer excellent spatial precision (i.e., the exact distances between the electrodes are known) and large brain areas in medial-lateral and anterior-posterior direction can be covered (**Figure 1Biv**, custom spacing between electrodes is in the range of 0.1–1 mm). This might be particularly advantageous for superficial recordings which are directed toward mapping neural responses in brain areas with large horizontal extensions (i.e., recordings from motor cortex). Further, several fibers can be included in one array (we tested up to two fibers per array) and the electrode number can be cranked up to 64 (we used up to 32 electrodes). From an economic perspective, however, the Microprobe-MEA are on the more expensive side. Further, although custom designs are offered, the turnaround is obviously not as quick as for in house manufactured devices. Together, directly comparing the optrode-MWA and the Microprobes-MEA, and also taken into account the better recording quality we obtained with the optrode-MWA (**Figures 5D,E**), we would suggest to start pilot experiments with the custom made device. For experiments which require exact knowledge of the electrode spacing, the Microprobe-MEA are obviously the device of choice.

The Optetrode drive (Anikeeva et al., 2012) allows advancing wires after implantation. This is particularly helpful for larger brain areas or structures which lie deeper within the brain as targeting becomes more difficult with depth. The drive is particularly light weight, thus even suitable for mice. For rats, the drive turned out to be too fragile, making a protective housing necessary. Depending on the task and recording environment, the material for the protective housing should be adapted (e.g., aluminum turned out to be a bad choice in our operant behavior chambers with metal walls). The targeting with the Optetrode drive is limited to one brain area per animal and the spacing precision between the 16 electrodes is limited and strongly depends on the skill set of the person who builds the drive (e.g., electrodes can be cut in a staggered manner by an experienced person), thus allowing targeting different depths simultaneously. The VersaDrive-8 Optical also allows the movement of electrode wires after implantation with the additional advantage of eight independently moveable bundles of four electrodes or one tetrode. Further, the fiber can be moved independently from all electrodes, adding further flexibility to adapt the recording and stimulation depth after the implantation. The drive is very compact and stable, thus ideally suited for rats which cannot rip the drive of with their hind paws. The spacing between the electrodes is defined by the placement of the guide tubes but the spacing between the electrodes within one guide tube is not known. So far, only one fiber can be included per drive and electrode bundles cannot be split up to 2 or more sides. Even though we did not find any significant improvement in recording quality when lowering the drive, the quality did not decrease over time (**Figure 5F**), and we were able to record more units per recording session for a longer period of time as compared to the fixed probes (**Figure 5E**). Further, we received a significantly higher recording quality compared to both tested fixed devices (**Figures 5D,E**).

In summary, we would recommend the custom-made optrode-MWA for pilot experiments or for simultaneously targeting multiple brain areas, the Microprobe-MWA for recordings in which the exact spacing between the electrodes has to be known and large brain areas need to be covered (e.g., in cortical mapping studies), the Optetrode drive for recordings in mice, and the Neuralynx VersaDrive-8 Optical for studies in rats in which it is crucial to measure for longer time periods and to advance wires and fiber over the course of the recordings.

## Ethics Statement

This study was carried out in accordance with the recommendations of the guidelines RL 2010 63 EU, Regierungspräsidium Darmstadt and the Regierungspräsidium Freiburg. The protocol was approved by the Regierungspräsidium Darmstadt and the Regierungspräsidium Freiburg.

## Author Contributions

SH and ID designed experiments. SH, MA, and ID wrote the manuscript. SH, MA, and DE performed experiments and analyzed data. DE programmed Matlab algorithm for automatic spike sorting and contributed to the electrophysiological recordings.

## Conflict of Interest Statement

The authors declare that the research was conducted in the absence of any commercial or financial relationships that could be construed as a potential conflict of interest.

## Footnotes

1 http://neuralynx.com/products/microdrives/versadrive_8_optical
